# Why is a functional PHO pathway required by fungal pathogens to disseminate within a phosphate-rich host: A paradox explained by alkaline pH-simulated nutrient deprivation and expanded PHO pathway function

**DOI:** 10.1371/journal.ppat.1007021

**Published:** 2018-06-21

**Authors:** Sophie Lev, Julianne Teresa Djordjevic

**Affiliations:** 1 Centre for Infectious Diseases and Microbiology, Westmead Institute for Medical Research, Westmead, Australia; 2 Sydney Medical School-Westmead, University of Sydney, Westmead, Australia; 3 Marie Bashir Institute for Infectious Diseases and Biosecurity, University of Sydney, Sydney, Australia; McGill University, CANADA

## Cross-species comparison of PHO signalling machinery reveals divergence at the transcriptional level

In metazoa, serum phosphate levels of approximately 1.18 mM are hormonally regulated to achieve equilibrium between extracellular phosphate absorption (via the intestine) and phosphate reabsorption from the kidney and bones [1, 2]. Consequently, cells are well supplied with phosphate and lack a PHO signalling network to maintain intracellular phosphate homeostasis. In contrast, phosphate acquisition is a major hurdle for fungi, which experience more extreme fluctuations in extracellular phosphate within their environment. Fungi have therefore evolved a tightly regulated PHO pathway to maintain intracellular phosphate homeostasis.

**Fig 1:**
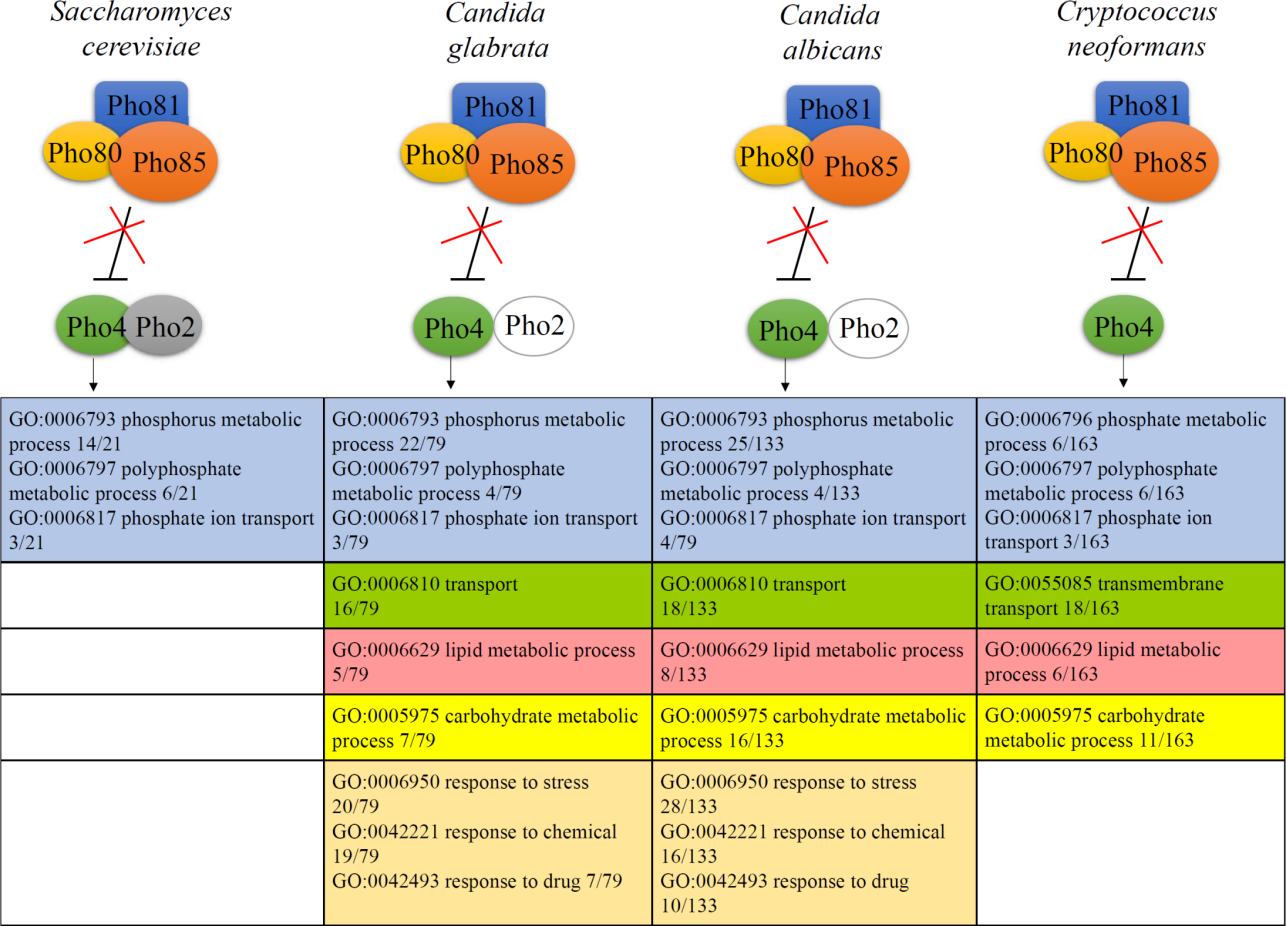
Gene ontology (GO) analysis of Pho4-dependent genes in *Saccharomyces cerevisiae*, *Candia albicans*, *Candida glabrata*, and *Cryptococcus neoformans*. The core regulatory machinery of the PHO pathway consisting of the CDK complex and the transcription factor Pho4 is conserved across the fungal species shown. However, evidence of Pho2 coregulation is absent in the fungal pathogens analysed. Pho4-dependent genes were assigned to GO groups with only the most abundant groups displayed in the analysis. Data for the comparison were assembled from Ogawa, 2000 (*S*. *cerevisiae*) [6]; Ikeh, 2016 (*C*. *albicans*) [7]; He, 2017 (*C*. *glabrata*) [8]; and Toh-e, 2015 (*C*. *neoformans*) [9]. GO annotation for *C*. *neoformans* JEC21 is publicly available at http://www.uniprot.org/uniprot/?query=jec21&sort=score and https://genome.jgi.doe.gov/Cryne_JEC21_1/Cryne_JEC21_1.home.html. Phosphate homeostasis-related genes in *C*. *neoformans* (blue) were manually annotated based on *S*. *cerevisiae* GO groups. GO groups in common are color coded. Note for *C*. *neoformans* that “transmembrane transport” is a child GO term for “transport” emphasizing the role of Pho4-dependent nutrient transport in this pathogen.

In *Saccharomyces cerevisiae*, phosphate status is conveyed to the nucleus via a core regulatory cyclin-dependent kinase (CDK) complex to affect a response at the transcriptional level (Fig 1). The CDK complex consists of the kinase Pho85 in association with the cyclin Pho80 and the CDK inhibitor Pho81. When phosphate is abundant, Pho85 phosphorylates transcription factor Pho4, thereby excluding it from the nucleus [3]. During phosphate deprivation, Pho81 inhibits Pho85. Nonphosphorylated Pho4 is subsequently retained in the nucleus where it triggers expression of genes involved in phosphate harvesting (PHO genes) in consort with the homeobox transcription factor Pho2 (reviewed in [4, 5]).

The mechanism of the phosphate starvation response in *S*. *cerevisiae* is essentially conserved in the nonpathogenic filamentous fungus *Neurospora crassa* and in the opportunistic yeast pathogens *C*. *albicans*, *C*. *glabrata*, and *C*. *neoformans*, except for some variation in the transcriptional regulator (Fig 1) [7–11]. In the *Candida* spp., Pho4 predominantly functions independently of Pho2, and in contrast to *S*. *cerevisiae*, Pho2-deficient *C*. *albicans* and *C*. *glabrata* still grow in the absence of phosphate [8]. In *C*. *neoformans* and *N*. *crassa*, there is no evidence that Pho4 (or its ortholog in *N*. *crassa*, Nuc-1 [10]) coregulates gene expression with other transcription factors, and no Pho2 ortholog has been identified, consistent with Pho4/Nuc-1 acting as the sole phosphate-responsive transcription factor. Furthermore, in contrast to CDK complex components, which are readily identified on the basis of homology with their counterparts in *S*. *cerevisiae*, Pho4 in *C*. *neoformans* was identified in mutant library screening for deficiency in acid phosphatase activity [9, 12]. In the nonpathogenic fission yeast *Schizosaccharomyces pombe*, response to phosphate starvation is mediated by the Zn_2_Cys_6_ binuclear cluster-containing transcription factor Pho7, which is a general stress-responsive transcription factor and structurally different to Pho4, which is a helix-loop-helix transcription factor [13].

## Absence of Pho4/Pho2 coregulation in *C*. *neoformans* and *Candida* spp. coincides with expansion of Pho4 gene targets

The ancestral function of the PHO pathway in fungi is to orchestrate induction of PHO genes in response to phosphate starvation. In *S*. *cerevisiae*, Pho4 and Pho2 coregulate the expression of approximately 20 PHO genes belonging to phosphate homeostasis-related GO groups (Fig 1, blue) [6]. In *C*. *glabrata* and *C*. *albicans* absence of Pho4/Pho2 combinatorial regulation coincided with a 4- and 7-fold expansion in PHO gene number to approximately 79 and approximately 133 genes, respectively [7, 8]. High-throughput analysis of Pho4-dependent genes in *C*. *neoformans* revealed an even greater expansion of the PHO gene repertoire to approximately 163 genes [9], once again coinciding with absence of Pho2 coregulation. Pho4 effectors in all 3 pathogens include the core group of genes encoding high-affinity phosphate transporters, acid phosphatases, phospholipid transporters, and the vacuolar polyphosphate polymerase [14, 15] (Fig 1, blue).

Comparison of Pho4 target genes not related to phosphate homeostasis in *C*. *neoformans* and *Candida* spp. reveal additional roles for the PHO pathway in cellular transport and in carbohydrate and lipid metabolism (Fig 1). Many of the genes in the transport category are involved in nutrient transport: in *C*. *neoformans*, 19 encode transmembrane proteins, including amino acid, calcium, siderochrome-iron, and sugar transporters. Thus, in contrast to *S*. *cerevisiae*, activation of the PHO pathway in fungal pathogens has the capacity to increase uptake of other nutrients in addition to phosphate. Furthermore, up-regulation of genes involved in carbohydrate and lipid metabolic processes may allow fungi to adapt their metabolism to survive within the host.

Interestingly, in contrast to *C*. *neoformans*, a large group of Pho4-dependent genes in the *Candida* spp. have functions associated with response to stress and chemicals, although genes in these stress-related categories differ in *C*. *albicans* and *C*. *glabrata*. This observation is consistent with Pho4 playing a role in stress tolerance in *Candida* spp. but not in *C*. *neoformans* [7, 12, 16, 17].

It should be noted that in nonpathogenic *N*. *crassa*, the PHO regulon also appears to be functionally expanded. In this fungus, the mitogen-activated protein kinase Mak-2 is thought to inhibit the cyclin–cyclin-dependent kinase complex to allow nuclear targeting of Nuc-1 during phosphate deprivation [18]. Utilizing a *mak2Δ* mutant, high-throughput analysis of gene expression demonstrated that out of 151 genes that are upregulated in the wild-type strain in a Mak-2–dependent manner during phosphate deprivation, many are involved in carbohydrate metabolism (GO:0005975) and transport (GO:0006810) and thus are similar to Pho4 targets in *C*. *neoformans* and *Candida* spp. [18]. A number of Nuc-1 effector genes that are not directly involved in phosphate homeostasis were identified in a suppression subtractive hybridization (SSH) study, consistent with the expanded PHO gene repertoire in *N*. *crassa* [19].

In *S*. *pombe*, the general stress-responsive transcription factor Pho7 binds within the promoters of genes involved in osmotic and nutrient stress response and the transport of nutrients other than phosphate (i.e., iron and copper). Each stress, including low phosphate, elicits a different Pho7–dependent transcriptional response. However, it appears that the main regulatory role of Pho7 is to coordinate stress-specific transmembrane transport [13].

Thus, in a number of fungal species, the response to phosphate deprivation involves activation of multiple processes and overlaps with the responses to other types of stress. It remains to be determined as to why Pho4–Pho2 regulates a phosphate starvation–specific pathway in *S*. *cerevisiae*, while in *S*. *pombe*, a general stress transcription factor Pho7 responds to phosphate deprivation.

He and colleagues [8] suggested that new niches encountered by *Candida* commensals in the host triggered evolution of new PHO pathway functions. However, given that PHO pathway expansion is evident in *C*. *neoformans*, which is a saprophyte rather than a commensal, environmental factors also drive PHO pathway evolution, an observation supported by functional expansion of the PHO regulon in *N*. *crassa* and *S*. *pombe* [13, 18].

## Fungal dissemination requires a functional and expanded PHO gene repertoire to overcome alkaline pH-simulated nutrient deprivation

The *pho4Δ* mutant in *C*. *neoformans* (*Cn*) shares phenotypic defects with *pho4Δ* in *S*. *cerevisiae (Sc)* and *C*. *albicans (Ca)* species backgrounds: reduced growth in low phosphate medium and at alkaline pH, including in medium designed to mimic physiological conditions (RPMI/5%C0_2_/pH7.4), irrespective of phosphate status [12]. Furthermore, *Cnpho4*Δ becomes susceptible to numerous stresses, including nitrosative stress, but only during phosphate deprivation. With the exception of alkaline pH, these stresses are rescued by phosphate replenishment [12]. Thus, stress sensitivity in *Cnpho4*Δ appears to be a secondary effect resulting from insufficient phosphate uptake. Alternatively, stress sensitivity could result from defective induction of Pho4-dependent genes required for stress tolerance in the absence of phosphate. In contrast, *Capho4*Δ is sensitive to cationic, alkaline, and oxidative stress independent of phosphate status, with deficiency in phosphate uptake correlating with disrupted metal ion homeostasis and altered copper bioavailability [7]. As copper is essential for superoxide dismutase (SOD) activity, oxidative stress susceptibility in *Capho4*Δ was attributed to compromised SOD activity [7]. Overall, the stress sensitivity differences in *Capho4*Δ and *Cnpho4*Δ are also reflected in the bioinformatics analysis (Fig 1).

Given that *C*. *neoformans* establishes a lung infection and then disseminates to the brain to cause meningitis, we tested *Cnpho4Δ* virulence in both murine inhalation and dissemination models. *CnPho4* was less virulent in the inhalation model, with growth in the lung reduced 10-fold relative to wild type (WT), and dissemination to the brain was almost abolished [12]. Remarkably, intravenous inoculation of *Cnpho4*Δ delayed mouse mortality by 30 days and significantly reduced lung and brain colonization as compared to WT. Although *Cnpho4*Δ exhibited some growth in lung postinhalation, there was no evidence of fungemia in either model [12]. *Capho4*Δ was also less virulent in a model of disseminated candidiasis, although fungemia was not assessed [7]. We conclude that fungal dissemination requires a functional PHO pathway.

Given that *Cnpho4Δ* is sensitive to alkaline pH, we reasoned that the stark difference between the growth of WT and *Cnpho4Δ* in tissue versus blood is pH-related. In the lung, *C*. *neoformans* cells proliferate rapidly in close proximity, forming cryptococcomas. The pH within the cryptococcoma is acidic (pH approximately 5.5) due to organic acid production [20, 21]. Thus, relative to WT, growth of *Cnpho4Δ* in the lung is reduced by only one order of magnitude. In contrast, individual fungal cells present in the blood during dissemination are dispersed by the circulation and are unable to lower the pH of this tightly buffered environment. Consequently, they experience alkaline pH stress, which we propose is overcome in WT but not in *Cnpho4Δ*, by activation of the PHO pathway, allowing phosphate uptake, proliferation, and colonization of the brain. Furthermore, alkaline pH-exposed *Cnpho4Δ* potentially becomes hypersusceptible to other host stresses, which could also negatively impact growth. Namely, the combination of alkaline pH stress and oxidative stress (for *Capho4*Δ) and nitrosative stress (for *Cnpho4Δ*) may be instrumental in allowing more efficient killing of these mutants by innate immune cells [7, 12].

An interesting question relates to the mechanism by which Pho4 allows fungi to tolerate alkaline pH. Similar to *S*. *cerevisiae*, *C*. *neoformans* is well adjusted to growing at acidic pH maintained by a membrane H^+^-ATPase, which pumps protons across the plasma membrane to establish a proton gradient. This gradient is essential for activity of H^+^ symporters involved in uptake of phosphate (Pho84 and Pho840) and other nutrients. Alkaline pH encountered during fungemia disrupts the protein gradient across the fungal membrane, simulating phosphate deprivation and compromising phosphate uptake. This would occur despite the >1-mM serum phosphate level, which is more than sufficient to suppress activation of the PHO pathway under acidic conditions [12]. There is evidence that phosphate uptake is compromised and that the PHO pathway is activated at alkaline pH: Pho4 translocates to nuclei in *S*. *cerevisiae* and *C*. *albicans*, following a pH shift from acidic to alkaline [22]; *pho4*Δ mutants in *S*. *cerevisiae*, *Candida* spp., and *C*. *neoformans* are sensitive to alkaline pH; PHO genes are induced in *S*. *cerevisiae* at alkaline pH in a Pho4-dependent manner [23]. Furthermore, Pho4 is required for induction of the phosphate transporters Pho84 and Pho89 in *C*. *neoformans* in minimal medium at alkaline pH and under physiological conditions, even when phosphate is abundant (RPMI/pH 7.4, 5.6 mM phosphate) [12]. Interestingly however, the *BTA1* gene, which is highly induced during phosphate deprivation in minimal medium under acidic conditions, is not induced in response to alkaline pH, suggesting that only a subset of Pho4 gene targets is induced under physiological conditions.

Evidence obtained by Kretschmer and colleagues demonstrates that phosphate transporters are not solely responsible for reduced dissemination of *pho4*Δ: a phosphate transporter–deficient mutant *Cnpho89*Δ*pho84*Δ*pho840* grew poorly during phosphate deprivation and was less virulent in a mouse inhalation model. However, in contrast to *Cnpho4*Δ, it still disseminated to the host brain [14]. Pho4-dependent activation of other gene(s) from the expanded Pho4 effector repertoire is therefore likely to mediate dissemination. Further studies are required to determine which of these genes are induced in response to alkaline pH and under physiological conditions. It is also possible that at alkaline pH, additional alkaline pH–responsive pathways, such as the Rim101 and cAMP/PKA pathways [24], cooperate with the PHO pathway to trigger expression of the selected PHO genes.

In summary, the paradoxical requirement for *C*. *neoformans* to up-regulate its PHO pathway in a phosphate-rich host can be explained by host alkaline pH simulating phosphate deprivation. Furthermore, expansion of the Pho4 gene repertoire provides the PHO pathway with the capacity to upregulate acquisition of nutrients other than phosphate and to effect carbohydrate and lipid metabolic processes instrumental in fungal metabolic adaptation to the nutrient environment within the host, particularly the blood.
